# Method for Measuring Phenotypic Colistin Resistance in Escherichia coli Populations from Chicken Flocks

**DOI:** 10.1128/AEM.02597-20

**Published:** 2021-02-12

**Authors:** Nguyen Thi Nhung, Nguyen Thi Phuong Yen, Nguyen Van Ky Thien, Nguyen Van Cuong, Bach Tuan Kiet, James Campbell, Guy Thwaites, Stephen Baker, Ronald B. Geskus, Juan Carrique-Mas

**Affiliations:** a Oxford University Clinical Research Unit, Ho Chi Minh City, Vietnam; b Sub-Department of Animal Health and Production, Cao Lanh, Dong Thap, Vietnam; c Centre for Tropical Medicine and Global Health, Nuffield Department of Clinical Medicine, Oxford University, Oxford, United Kingdom; d Cambridge Institute of Therapeutic Immunology & Infectious Disease, University of Cambridge, Cambridge, United Kingdom; Centers for Disease Control and Prevention

**Keywords:** poultry, colistin resistance, broth microdilution, colistin use

## Abstract

Colistin is extensively used in animal production in many low- and middle-income countries. There is a need to develop methods to benchmark and monitor changes in resistance among mixed commensal bacterial populations in farms. We aimed to evaluate the performance of a broth microdilution method based on culturing a pooled Escherichia coli suspension (30 to 50 organisms) obtained from each sample. To confirm the biological basis and sensitivity of the method, we cultured 16 combinations of one colistin-susceptible and one *mcr-1*-carrying colistin-resistant E. coli isolate in the presence of 2 mg/liter colistin. Readings of optical density at 600 nm (OD_600_) over time were used to generate a growth curve, and these values were adjusted to the values obtained in the absence of colistin (adjusted area under the curve [AUC_adj_]). The median limit of detection was 1 resistant in 10^4^ susceptible colonies (1st to 3rd quartile, 10^2^:1 to 10^5^:1). We applied this method to 108 pooled fecal samples from 36 chicken flocks from the Mekong Delta (Vietnam) and determined the correlation between this method and the prevalence of colistin resistance in individual colonies harvested from field samples, determined by the MIC. The overall prevalences of colistin resistance at the sample and isolate levels (estimated from the AUC_adj_) were 38.9% (95% confidence intervals [CI], 29.8 to 48.8%) and 19.4% ± 26.3% (± values are standard deviations [SD]), respectively. Increased colistin resistance was associated with recent (2 weeks) use of colistin (odds ratio [OR] = 3.67) and other, noncolistin antimicrobials (OR = 1.84). Our method is a sensitive and affordable approach to monitor changes in colistin resistance in E. coli populations from fecal samples over time.

**IMPORTANCE** Colistin (polymyxin E) is an antimicrobial with poor solubility in agar-based media, and therefore, broth microdilution is the only available method for determining phenotypic resistance. However, estimating colistin resistance in mixed Escherichia coli populations is laborious, since it requires individual colony isolation, identification, and susceptibility testing. We developed a growth-based microdilution method suitable for pooled fecal samples. We validated the method by comparing it with individual MIC determinations for 909 E. coli isolates; we then tested 108 pooled fecal samples from 36 healthy chicken flocks collected over their production cycle. A higher level of resistance was seen in flocks recently treated with colistin in water, although the observed generated resistance was short-lived. Our method is affordable and may potentially be integrated into surveillance systems aiming at estimating the prevalence of resistance at colony level in flocks/herds. Furthermore, it may also be adapted to other complex biological systems, such as farms and abattoirs.

## Abstract (precis)

Colistin (polymyxin E) is an antimicrobial with poor solubility in agar-based media, and therefore, broth microdilution is the only available method for determining phenotypic resistance. However, estimating colistin resistance in mixed Escherichia coli populations is laborious, since it requires individual colony isolation, identification, and susceptibility testing.

## INTRODUCTION

Colistin (polymyxin E) is a last-resort drug used for the treatment of severe multidrug-resistant (MDR) infections in many countries and is classified by the World Health Organization (WHO) as a “highest priority, critically important” antimicrobial (1). The emergence of *mcr-1* plasmid-encoded colistin resistance among Gram-negative bacteria is considered a serious threat to global health (2). It has been hypothesized that colistin use in animal production is a major contributing factor to the emergence of colistin resistance worldwide (3). Colistin is still used in poultry and pig farming in many countries (4). In terms of frequency, colistin is the most commonly used antimicrobial in chicken production in the Mekong Delta region of Vietnam (5, 6). Studies in the same region have shown that resistance against colistin in commensal Escherichia coli from chicken flocks is often encoded by the *mcr-1* gene (7, 8). At sample level, the prevalence of *mcr-1* in chicken fecal samples in the Mekong Delta was 59.4%. The prevalence of this gene has also been found to be higher among in-contact humans (chicken farmers) than in individuals in urban areas (7).

E. coli is an ubiquitous commensal enteric organism globally used to monitor phenotypic antimicrobial resistance (AMR) in national surveillance programs, both in humans and in animals (9, 10). Given the diversity of this organism within the enteric microbiome, the characterization of phenotypic resistance in a mixed population of commensal E. coli requires selecting a representative and sufficiently large number of strains. This is often achieved by performing differential colony counts on agar media with and without antimicrobials (11). However, agar-based methods are not appropriate for colistin given the antimicrobial’s poor diffusion (12). Determination of the MIC by broth microdilution is regarded as the gold standard for testing of colistin resistance of *Enterobacteriaceae* (ISO 20776-1) both by the Clinical and Laboratory Standards Institute (CLSI) and the European Committee on Antimicrobial Susceptibility Testing (EUCAST) (12, 13). Establishing accurately the prevalence of resistance at the colony level requires the investigation of a sufficiently large, representative number of isolates from each sample, which is extremely laborious and costly (8, 11, 14). Therefore, there is a need for cost-effective methods for evaluating resistance against colistin in mixed E. coli populations from animal fecal samples. Here, we designed and evaluated a broth microdilution-based method to quantify colistin resistance in E. coli populations from pooled chicken fecal samples. We then related the observed results to data on antimicrobial use (AMU) from the same flocks.

## RESULTS

### Growth of standard suspensions.

**FIG 1:**
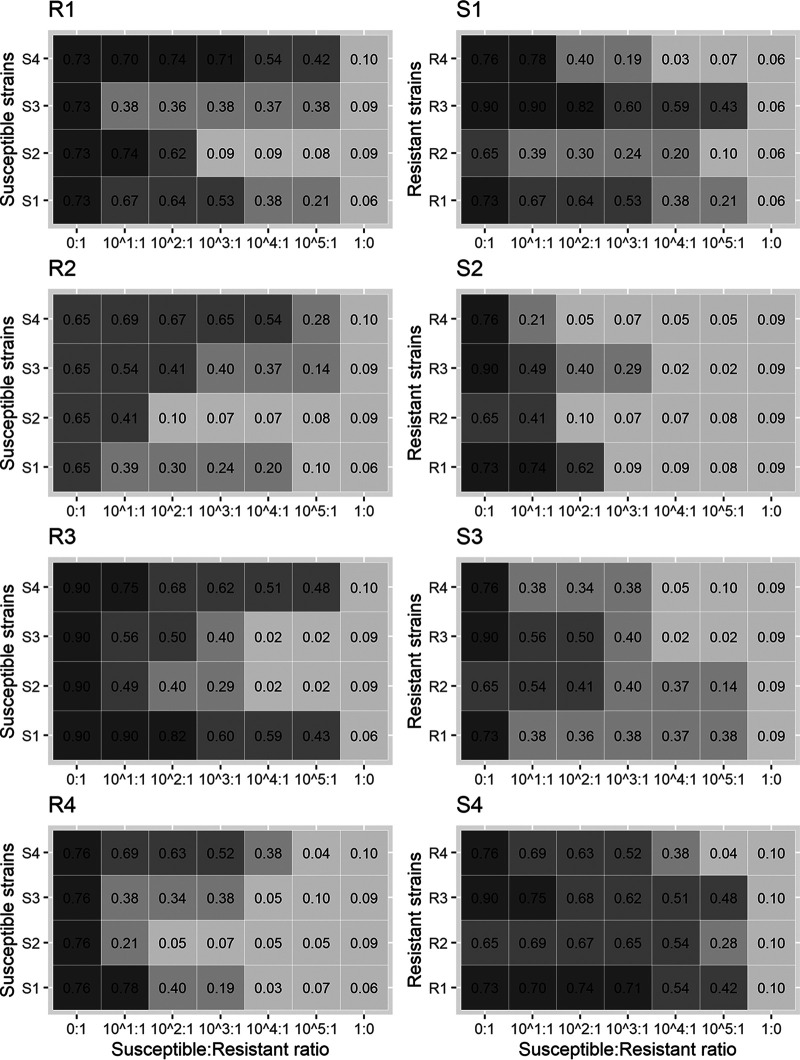
AUC_adj_ of standard suspensions. Positive growth values are represented by increasing strength of color. R, resistant; S, susceptible. The average AUC_adj_ values for strains R1, R2, R3, and R4 were 0.40, 0.30, 0.41, and 0.26, respectively. Average AUC_adj_ values for strains S1, S2, S3, and S4 were 0.41, 0.19, 0.31, and 0.54, respectively.

The adjusted area under the curve (AUC_adj_) values generated from all susceptible-resistant strain combinations are presented in Fig. 1. Based on the AUC_adj_ value obtained with susceptible strains (0.09 ± 0.02; ± values are standard deviations [SD]), we considered any sample with an AUC_adj_ of >0.13 positive for colistin resistance. In all cases, AUC_adj_ values increased with increasing ratio of resistant to susceptible organisms. Growth was detected at maximum ratios of susceptible (S) to resistant (R) strains of 10^5^:1, 10^4^:1, 10^3^:1, 10^2^:1, and 10^1^:1 for 43.7%, 12.5%, 18.5%, and 12.5% and 12.5% of combinations, respectively. There was no difference in average AUC_adj_ between resistant strains with low (R1 and R2; colistin MIC = 4 mg/liter) and moderate (R3 and R4; colistin MIC = 8 mg/liter) levels of resistance (both AUC_adj_ = 0.39; Kruskal Wallis test, *P* = 0.688). The observed variation in AUC_adj_ values depended on the choice of resistant and susceptible strains. In combinations with resistant strains, S2 yielded the lowest average AUC_adj_ (median, 0.09 [1st to 3rd quartile, 0.07 to 0.29]) as well as the lowest limit of detection (average S-R ratio of 10^2^:1), whereas S4 gave the highest AUC_adj_ (median 0.62 [1st to 3rd quartile, 0.48 to 0.69]) as well as the highest limit of detection (average S-R ratio of 10^5^:1).

### Study flocks and their AMU.

A total of 36 flocks (108 samples) were investigated in this study. The median flock size was 231 (1st to 3rd quartile, 189 to 401) chickens. Flocks were raised over a median of 19 (1st to 3rd quartile, 17 to 20) weeks. Colistin had been administered to 22/36 (61.1%) flocks. Among flocks given colistin, the average number of animal daily doses (ADD) per 1,000 chicken-days of this antimicrobial administered over the production cycle was 149.5 ± 261.6. Colistin was used more during the early flock cycle period (281.7 ± 321.2 ADD per 1,000 chicken-days) compared with the second period (17.4 ± 18.1 ADD per 1,000 chicken-days) (Wilcoxon paired test, *P* < 0.001) (Table 1). This antimicrobial was administered over a median of 4 (1st to 3rd quartile, 2 to 6) weeks. The data for colistin use among study flocks are displayed in Fig. S1 in the supplemental material.

**TABLE 1:** Description of AMU and estimated prevalence of colistin resistance in 36 small-scale chicken flocks stratified by colistin administration

Parameter	Value for flocks		
	Not using colistin (*n* = 14)	Using colistin (*n* = 22)	All flocks (*n* = 36)
Cycle duration (wks) [median (1st–3rd quartile)]	19 (17–20)	20 (17–21)	19 (17–20)
No. of chickens [median (1st–3rd quartile)]	249 (194–482)	208 (128–398)	231 (189–401)
No. of ADD of colistin (per 1,000 chicken-days) (mean ± SD)			
First period	0	281.7 ± 321.2	172.1 ± 285.1
Second period	0	17.4 ± 18.1	10.6 ± 16.4
Whole production cycle	0	149.5 ± 261.6	91.4 ± 216.4
No. of ADD of noncolistin antimicrobials (per 1,000 chicken-days) (mean ± SD)			
First period	345.5 ± 471.5	629.3 ± 359.8	518.9 ± 424.2
Second period	29.0 ± 48.6	72.5 ± 98.5	55.6 ± 84.7
Whole production cycle	187.2 ± 366.2	350.9 ± 383.8	287.3 ± 382.9
No. of flocks using colistin 2 wks prior to:			
Midproduction sampling	0	11	11
End-of-production sampling	0	1	1
AUC_adj_ [median (1st–3rd quartile)]			
In day-old chicks	0.07 (0.04–0.42)	0.06 (0.04–0.52)	0.07 (0.04–0.65)
At midproduction	0.06 (0.03–0.43)	0.54 (0.07–0.65)	0.20 (0.05–0.63)
At end of production	0.07 (0.06–0.55)	0.07 (0.06–0.55)	0.07 (0.05–0.56)
Prevalence of resistance (%) at sample level (95% CI)			
In day-old chicks	42.8 (18.8–70.3)	31.8 (14.7–54.9)	36.1 (21.3–53.8)
At midproduction	28.6 (9.5–58.0)	63.6 (40.8–82.0)	50.0 (34.5– 65.5)
At end of production	28.6 (9.5–58.0)	31.8 (14.7–54.9)	30.5 (16.9– 48.3)
Estimated prevalence of resistance (%) at colony level (mean ± SD)			
In day-old chicks	28.8 ± 36.0	15.7 ± 24.7	20.8 ± 29.8
At midproduction	17.3 ± 28.7	27.0 ± 26.4	23.3 ± 27.4
At end of production	16.2 ± 24.8	12.8 ± 18.1	14.1 ± 20.7

^a^ AUC, area under the growth curve; CI, confidence interval; SD, standard deviation.

In addition to colistin, a total of 27 noncolistin antimicrobials (belonging to 12 classes) were administered to study flocks. In decreasing order, oxytetracycline, tylosin, neomycin, ampicillin, streptomycin, and doxycycline were the antimicrobials most used. The average number ADD per 1,000 chicken-days of other antimicrobials among flocks using colistin was higher than flocks that did not use colistin (350.9 ± 383.8 versus 187.2 ± 366.2; Wilcoxon test, *P* = 0.004). Among both type of flocks, antimicrobials were administered more commonly during the first period (average number of ADD per 1,000 chicken-days, 629.3 ± 359.8 and 345.5 ± 471.5, respectively) than the second period of chicken life (average number of ADD per 1,000 chicken-days, 72.5 ± 98.5 and 29.0 ± 48.6, respectively) (Table 1). The frequencies of use of noncolistin antimicrobials in the flocks studied are presented in Table S1.

### Prevalence of colistin resistance at the colony level.

**FIG 2:**
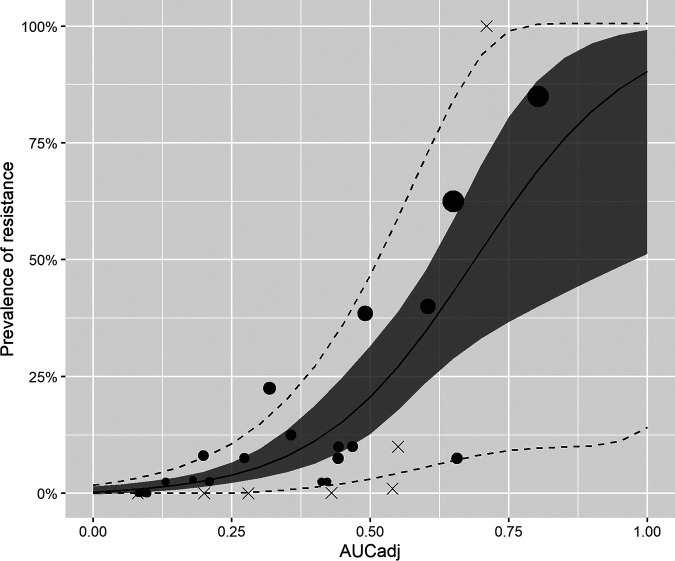
Relationship between AUC_adj_ (from a mix of 40 E. coli isolates per sample) and prevalence of colistin resistance at the colony level. The figure shows the predicted mean value of resistance with the pointwise 95% confidence interval (shaded area). The dotted lines give the 5% and 95% prediction intervals. Circles indicate AUC_adj_ values for mixed E. coli isolates in field samples. The size of the dot represents the average MIC of each sample. Multiplication signs indicate AUC_adj_ values for mixed susceptible and resistant strains.

A total of 909 E. coli strains were isolated from 23 selected samples (∼40 E. coli isolates/sample) and were tested for their MIC against colistin. Among those, total of 129 strains (14.2%) were resistant to colistin. Of resistant strains, 75.2% strains had a MIC of 4 mg/liter, whereas 24.0% had a MIC of 8 mg/liter. Only 1 isolate (0.8%) displayed a MIC of 16 mg/liter (Fig. S2). The beta-regression model that relates the AUC_adj_ (obtained from suspensions of 40 E. coli strains) to the percentage of resistant E. coli strains is shown in Fig. 2. The trend over AUC_adj_ was highly significant (*P* < 0.001). The equation 100/(1 + *e*^4.8−(7.04×AUCadj)^) associated with this model was applied for estimating the prevalence of colistin resistance at colony level among field samples.

### Changes in AUC_adj_ over the production cycle and prevalence of colistin resistance.

Overall, there was no significant change in colistin resistance (AUC_adj_) over the production cycle (*P* = 0.569) (Fig. S3). Among flocks not exposed to colistin (*n* = 14), the differences in AUC_adj_ between sampling points were small. However, among flocks using colistin (*n* = 22), the AUC_adj_ values for midproduction samples (0.54 [1st to 3rd quartile, 0.07 to 0.65]) were higher than those for samples from day-old chicks (0.06 [1st to 3rd quartile, 0.04 to 0.52]) (Wilcoxon paired test, *P* = 0.063) and end-of-production samples (0.07 [1st to 3rd quartile, 0.06 to 0.55]) (Wilcoxon paired test, *P* = 0.046). There was little or no difference in AUC_adj_ values between samples from day-old chicks and end-of-production samples (Table 1).

The prevalence of colistin resistance at sample level was 38.9% (95% confidence interval [CI], 29.8 to 48.8%) (42/108 positive samples). The prevalences of resistance in day-old-chick, midproduction, and end-of-production samples were 36.1%, 50.0%, and 30.5%, respectively (χ^2^ test, *P* = 0.219). The overall average estimated prevalence of resistance at the colony level (generated from AUC_adj_) was 19.4% ± 26.3%. Among flocks using colistin, the highest level of resistance corresponded to midproduction samples (27.0% ± 26.4%), followed by day-old-chick samples (15.7% ± 24.7%) and end-of-production samples (12.8% ±1 8.1%) (Kruskal Wallis test, *P* = 0.070). In contrast, among flocks not using colistin, day-old-chick samples showed higher prevalence of resistance (28.8% ± 36.0%) than those from midproduction (17.3% ± 28.7%) and the end of production (16.2% ± 24.8%) (Kruskal Wallis test, *P* = 0.453). Summary results are presented in Table 1, and results for individual samples are given in Table S2.

### Risk factors for colistin resistance.

Table 2 shows results for univariable and multivariable analyses. In the multivariable model, use of colistin during the 2 weeks prior to sampling (odds ratio [OR] = 3.67; 95% CI = 0.68 to 19.7) and use of noncolistin antimicrobials (OR = 1.84; 95% CI = 0.88 to 3.85) were associated with colistin resistance at the sample level.

**TABLE 2:** Logistic regression models investigating risk factors associated with colistin resistance in chicken flocks at sample level^*a*^

Variable	Univariable^*b*^			Multivariable			
	OR	95% CI	*P* value	OR	95% CI	*P* value	
Age of chicken flock (wks)	0.93	0.84–1.02	0.156	1.04	0.91–1.18	0.605
Use of colistin within last 2 wks (yes/no)	5.30	1.17–24.08	0.030	3.67	0.68–19.70	0.128
No. of ADD per 1,000 chicken-days of colistin^*c*^	1.66	1.00–2.76	0.049	1.06	0.55–2.06	0.845
Colistin resistance of day-old chicks (yes/no)	1.45	0.53–3.97	0.461	1.61	0.54–4.84	0.395
No. of ADD per 1,000 chicken-days of noncolistin antimicrobials^*c*^	2.10	1.18– 3.73	0.012	1.84	0.88–3.85	0.102

a Models were based on a total of 72 samples (midproduction and end of production); 29 were positive for resistance to colistin. ADD, animal daily dose; OR, odds ratio; CI, confidence interval.

b The age of the chicken flock was included as a variable in all univariable models to calculate estimates for all subsequent variables.

c Logarithmically transformed after adding 1.

### Estimation of test costs.

The reagent and medium costs of broth microdilution and Etest for testing one sample based on the investigation of 10 E. coli isolates were ∼25 and ∼63 U.S. dollars (USD), respectively. The cost for testing one sample by the growth-based method (based on 40 isolates) was ∼6.5 USD. In addition, broth microdilution involved a higher labor cost (average of ∼1 person-day per sample) than either the Etest or the growth-based method (∼0.5 person-day) (Table S3).

## DISCUSSION

Here, we developed a method that may be effectively used to quantify colistin resistance in commensal E. coli in chicken flocks. Colistin is widely used in poultry and pig production worldwide (4, 15, 16). In the Mekong Delta of Vietnam, colistin is typically administered to chicken flocks in drinking water during the brooding period (1 to 4 weeks) with a prophylactic purpose (i.e., to prevent disease) (5). Colistin is also included in some pig and poultry commercial feeds as an antimicrobial growth promoter (AGP) (17). However, from 2020 onward, AGPs are longer allowed in Vietnam (law no. 32/2018/QH14), in line with legislative restrictions in Thailand (2015) (18), China (2016) (19), and India (2019) (20).

In contrast with the study of human patients, where colistin susceptibility testing is required to inform therapeutic choices (21), our method is aimed at estimating colistin resistance in mixed commensal E. coli populations. Through evaluation of the growth curves of standard E. coli suspensions from fecal samples, our method enables the detection of colistin resistance in a dichotomous fashion (presence/absence), as well as providing a quantitative assessment of colistin resistance at the colony level (prevalence of resistant E. coli). The sensitivity of this method is, however, limited by the number of colonies harvested per sample (30 to 50) and may therefore miss colistin-resistant strains in situations of very low prevalence. Indeed, statistically, given a sample of 40 colonies, there is a 5% probability of not detecting colistin resistance in any of them when the prevalence of resistant falls below 7.5%. Because of this, the method is more suitable for situations of medium to high prevalence of colistin resistance. The sensitivity could, however, be potentially increased by collecting several samples or increasing the number of E. coli colonies used in each suspension. For example, detection of a prevalence of 2% would require the investigation of 150 isolates (∼4 samples, each with 30 to 50 colonies), while detection of a prevalence of 1% would require 300 isolates (∼8 samples), and detection of 0.1% prevalence would require a total of 3,000 isolates (∼75 samples).

Although there was a statistically significant correlation between the prevalence of resistance and AUC_adj_, we observed considerable variation in AUC_adj_ for similar prevalence values both in our laboratory validation and in our flock samples. This suggests variable growth capacity among resistant strains, which may depend on their relative fitness. In the case of field suspensions containing a diversity of susceptible and resistant strains, it is also likely that the relative composition of strains may result in variable growth among the resistant strains due to the liberation of bacteriocin (i.e., colicins) in the culture medium (22) or the presence of bacteriophages. This may also explain the variable limit of detection confirmed in laboratory conditions with different susceptible strains. In general, given identical prevalence of resistant strains, we observed higher AUC_adj_ values for individual susceptible-resistant strain combinations than for the specific mix of E. coli isolates in field samples (Fig. 2). This could be probably explained by less competition exerted in mixes containing a single strain, compared with heterogenous mixes containing ∼40 different strains. Because of these reasons, prevalence estimates derived from AUC_adj_ should always be interpreted with caution.

We believe that our testing approach is more efficient than isolating and investigating individual colonies, as it entails a lower cost. However, it requires investment in a microplate reader costing between 3,000 and 10,000 USD. The technique presented here could potentially be adapted to the investigation of other types of phenotypic resistance in E. coli (i.e., tetracycline, ampicillin, etc.), but it would necessarily require optimizing working concentrations.

At the colony level, we obtained a median prevalence of 19.4% colistin resistance in flocks. These results are comparable with previous studies on chicken E. coli isolates in the area (12 to 22%) (7, 8). Furthermore, the observed ∼40% resistance at the sample level is consistent with a previous study on chickens in the Mekong Delta of Vietnam, where 5 E. coli colonies were investigated from each of 18 fecal samples (8). In that study, a total of 8/18 (44%) samples included at least one resistant strain (N. T. Nhung, unpublished data). A PCR-based study in this region reported that 59.4% of chicken samples investigated tested positive for the *mcr-1* gene (7).

We demonstrated a short-term increase in phenotypic colistin resistance following administration of colistin use as well as noncolistin antimicrobials. This contrasts with a study conducted on a broiler flock in France, where administration of colistin failed to induce colistin resistance in *Enterobacteriaceae* (including E. coli) (23). However, unlike in Vietnam, colistin use and resistance (including *mcr-1*) are relatively rare in European livestock (10). Overall, we found relatively high levels of colistin resistance (∼40%), even in flocks that had not been given colistin (33.3%). There was evidence of colistin resistance in midproduction samples from flocks that had previously tested negative in day-old-chick samples and had not been administered colistin (3 of 8 flocks) (data not shown). This suggests that colistin resistance may have been generated in or introduced into study flocks from other sources, such as contaminated water or feed, or due to contamination with bacteria from other animal species present in these small-scale farms.

Our findings of increased colistin resistance in flocks treated with antimicrobials other than colistin are intriguing. In a previous study on Mekong Delta pig farms, colistin resistance in E. coli strains was associated with use of noncolistin antimicrobials such as quinolones and cephalosporins (8). The presence of genes conferring resistance against several different antimicrobial classes in *mcr*-harboring plasmids may explain these findings and suggest that the use of noncolistin drugs may also select for colistin resistance (24).

We observed a peak of colistin resistance in midproduction samples among flocks using colistin, and levels of resistance generally decayed subsequently. This is likely to reflect the higher frequency of colistin use during the brooding period. A longitudinal study on travelers colonized by *mcr-1*-carrying bacteria showed that they were able to completely eliminate these bacteria within 1 month after returning to their home country (25). The reasons for a reduction in resistance over time are unknown, and it may be due to a combination of factors leading to plasmid loss and/or fitness costs. However, studies in the laboratory have shown that the presence of plasmid-mediated colistin resistance has been shown to entail no fitness costs for E. coli (26). It is worth noting that in our study, chicken flocks were of a local native breed, and they were typically raised over a 4- to 5-month period, a period much longer than that required by industrial broilers (typically 1.5 months). This suggests that birds slaughtered earlier may have a higher prevalence of colistin resistance, and this potentially represents an additional risk to the consumer.

In summary, our method may be adapted to benchmark and monitor changes over time in colistin resistance in fecal samples in other complex biological systems, such as abattoirs, slaughter points, and sewage, or even in human individuals. Our results indicate a high background of colistin resistance even in flocks not given this antimicrobial. The observed increases after colistin use were short-lived and suggest that in small-scale farming systems, reducing colistin resistance may require increasing biosecurity as well as restocking colistin-negative day-old chicks.

## MATERIALS AND METHODS

### Study design.

In order to investigate the biological basis and the limit of detection of the proposed method, we used four previously characterized *mcr-1* colistin-resistant E. coli strains, two displaying moderate-level (MIC = 8 mg/liter) and two low-level (MIC = 4 mg/liter) colistin resistance, alongside four colistin-susceptible strains. We prepared standard bacterial suspensions consisting of a mix of each of the resistant and the susceptible strains at different ratios; these were incubated in medium with and without 2 mg/liter of colistin. A growth curve from each suspension was obtained by measuring the optical density at 600 nm (OD_600_) during incubation. The area under the curve (AUC_adj_) of each colistin-containing standard suspension was adjusted by the AUC values obtained from its equivalent colistin-free suspension. We investigated the relationship between the prevalence of resistance at colony level and the observed AUC_adj_ from the examination of 30 to 50 individual E. coli isolates from each of 23 samples and obtained a model equation. We calculated AUC_adj_ values of suspensions consisting 30 to 50 E. coli colonies harvested from each of 108 pooled fecal samples from 36 small-scale (single-age) chicken flocks raised in Dong Thap province (Mekong Delta, Vietnam) (27). We inferred the prevalence of resistant E. coli in flock samples investigated by extrapolation using the model equation. The contribution of colistin use and other antimicrobials administered to flocks on the observed phenotypic colistin resistance was investigated by building logistic regression models with age as the primary time variable.

### Culture of standard suspensions and calculation of the AUC_adj_ and limit of detection.

Each of the chosen resistant E. coli strains (named R1 to R4, where R1 and R2 had MICs of 4 mg/liter and R3 and R4 had MICs of 8 mg/liter) and susceptible strains (S1 to S4, all with MICs of ≤1 mg/liter) was incubated in cation-adjusted Mueller-Hinton II broth (MHB2; Sigma-Aldrich, USA) at 37°C and 200 rpm for 4 h (log phase). Bacterial inocula were adjusted to 10^8^ CFU/ml (OD_600_ = 0.1) and then diluted with MHB2 to 10^6^ CFU/ml. Each susceptible strain was mixed with a resistant strain, giving a total of 16 combinations with susceptible-resistant ratios ranging from 1:0 (susceptible strain only) to 0:1 (resistant strain only). Intermediate ratios were 10^1^:1, 10^2^:1, 10^3^:1, 10^4^:1, and 10^5^:1. A total of 100 µl of each suspension was added into a well of a polystyrene microplate (Corning, USA) containing 100 µl of colistin solution (final working concentration was 2 mg/liter). In addition, respective colistin-free (control) suspensions were prepared. Plates were incubated in a microplate reader (SpectroStar; BMG Labtech, Germany) at 37°C for 20 h, and the turbidity (OD_600_) readings were recorded every hour. All experiments were conducted in triplicate.

The areas under the curves (AUC) generated over the 20-h observation period were computed. The AUC value generated from each standard suspension (AUC*_i_*) was related to the AUC generated by its respective colistin-free control (AUC_adj_ = AUC*_i_*/AUC_0_). Samples with AUC_adj_ values greater than the average value obtained with each of the four susceptible strains plus 2 SD were considered positive for colistin resistance.

### Flock sample and AMU data collection.

Fresh pooled fecal samples were collected from each flock at three time points: (i) day-old chicks, (ii) ∼2- to 3-month-old chicks (midproduction), and (iii) ∼4- to 6-month-old chicks (end of production). Day-old-chick fecal (i.e., meconium) samples were collected from the crates at the time when chicks were delivered to the farms. For midproduction and end-of-production sampling, sterile paper liners were placed near drinkers and feeders in the chicken house/pen to collect deposited droppings. After a minimum of 10 droppings had been deposited, liners were swabbed using sterile gauze. Each collected gauze was placed in a universal jar and mixed vigorously with 50 ml saline buffer. One milliliter of the resulting eluate was stored at −20°C with glycerol. Data on AMU had been collected using diaries designed for this purpose, where farmers were asked to note all antimicrobials used. Farmers were instructed to keep all packages of antimicrobials used in their flocks (5). Sample and data collection were conducted between October 2016 and October 2018.

### Testing of pooled fecal samples.

Eluates from pooled fecal samples were plated on ECC agar (CHROMagar, France) and incubated at 37°C for 20h. A total of 30 to 50 E. coli (blue) colonies from each agar sample were picked, pooled, and incubated in cation-adjusted MHB to log phase. The resulting bacterial suspensions were investigated as described above.

### Estimation of the prevalence of colistin resistance at the colony level.

We selected a number of positive samples with variable levels of AUC_adj_. For each sample, 40 E. coli isolates were obtained and tested individually for colistin MIC by standard broth microdilution. These pools of 40 E. coli were also investigated for their AUC_adj_ as described above.

### Data analyses and cost estimation.

In order to relate the AUC_adj_ value to the measured prevalence of resistance among selected samples, we fitted a beta-regression model using the betareg package in R (28). Both the trend and the dispersion were allowed to vary over AUC_adj_ in a linear way.

AMU in flocks was quantified for the two periods defined by the sampling schedule: (i) between restocking and midproduction and (ii) between midproduction and end of production. Weekly estimates of colistin use were expressed as the number of ADD (animal daily doses administered per 1,000 chicken days) calculated for each of the two periods (5). Risk factors associated with colistin resistance at midproduction and end of production were investigated by logistic regression. The outcome was colistin resistance (yes/no) at the sample level. The variables investigated were (i) age of chicken flock (weeks), (ii) use of colistin within 2 weeks prior to sampling (yes/no), (iii) number of ADD per 1,000 chicken-days of colistin in each period, (iv) colistin resistance of day-old chicks (yes/no), and (v) number of ADD per 1,000 chicken-days of noncolistin antimicrobials used in each period. The age of the chicken flock was included as a variable in all univariable models because it is the principal time variable. Since we had two measurements per flock (midproduction and end-of-production samples), we used generalized estimation equations with an exchangeable correlation structure to estimate the parameters using the R package geepack (29, 30).

The change in AUC_adj_ over age of chicken was modeled using a random-effects linear regression. In order to allow for a nonlinear trend, we used a natural spline for the fixed effect term (knots at 0, 8, 12, and 20 weeks). We allowed for a random intercept and linear trend by age.

The overall costs (per sample) of the method described above were calculated based on expenditures on medium, reagents, and consumables (excluding staff time, which was estimated separately). The estimated costs were compared with those incurred in testing one sample by broth microdilution and Etest in Vietnam as of January 2020. Our calculations were based on the investigation of 40 E. coli isolates per sample using the growth-based method, compared with 10 isolates each by broth microdilution and by Etest.

## Supplementary Material

Supplemental file 1

Supplemental file 2

Supplemental file 3
