# Supplementary material for: Method for Measuring Phenotypic Colistin Resistance in Escherichia coli Populations from Chicken Flocks
Source: Appl Environ Microbiol. 2021 Feb 12;87(5):e02597-20. doi: 10.1128/AEM.02597-20 (PMC8090885; doi:10.1128/AEM.02597-20)
Supplement: Supplemental file 1 [file AEM.02597-20-s0001.pdf]

Table S3. Estimated costs (in US dollars) (USD) incurred in testing one faecal sample to determine phenotypic colistin resistance in commensal *E. coli*.

Item	Broth microdilution (10 strains)	Etest (10 strains)	Pooled growth method (1 pool of 40 strains)*
Isolation	4.5	4.5	2
Antimicrobial reagent	15	50	1.5
Mueller Hinton broth media	1	NA	0.1
Mueller Hinton agar	NA	5	NA
Consummables	4	3	3
**Total (USD) (excluding labour)**	**24.5**	**62.5**	**6.6**
Labor cost (person-days)	1	0.5	0.5

*Costs related to testing four different colistin concentrations. NA= not applicable.

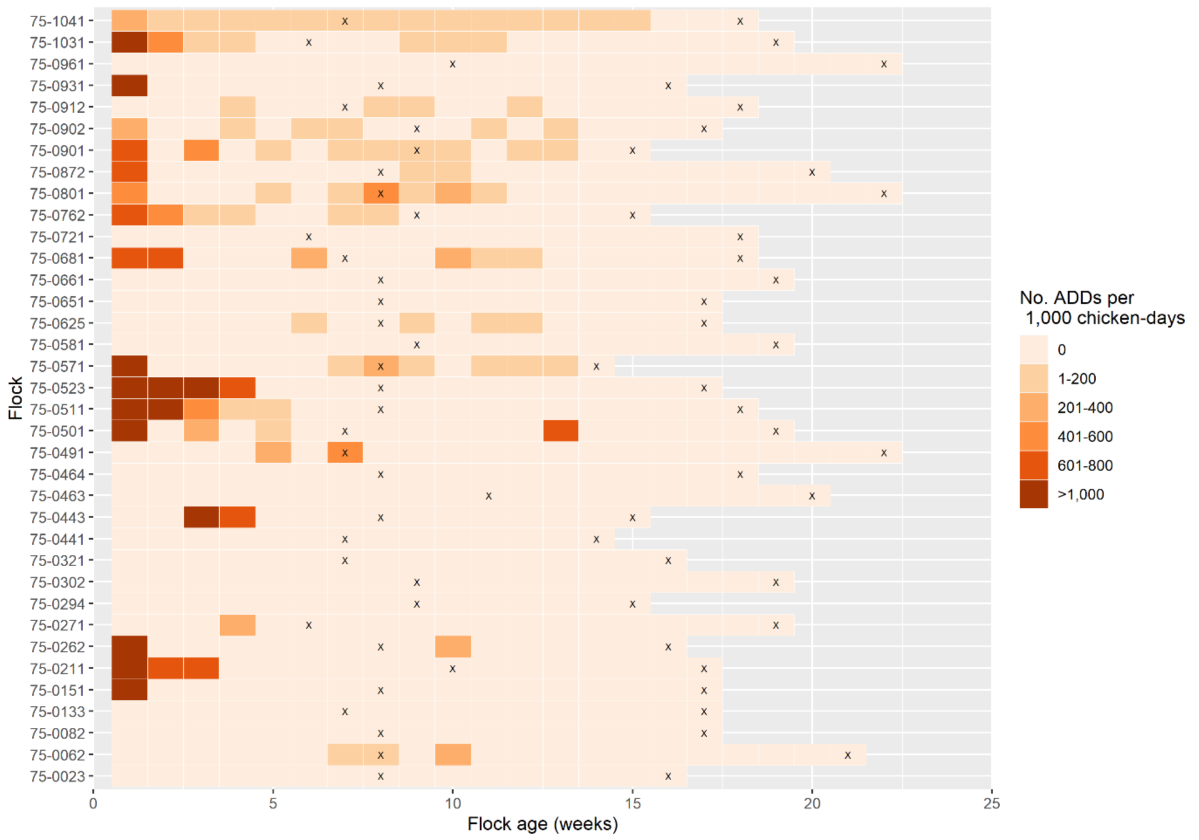

FIG S1 Usage of colistin among study flocks by week. Each line represents one flock and each column represents one week. The crosses indicate week of sampling. Rectangles indicate the number of Animal Daily Doses (ADD) of colistin administered per 1,000 chicken days in each flock.

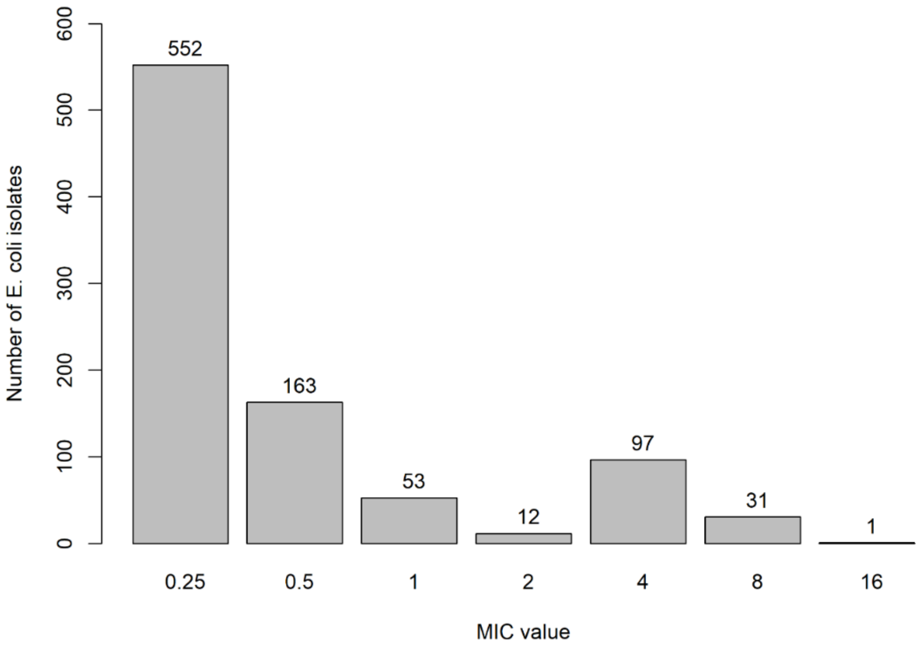

FIG S2 Distribution of MIC values among 909 *E. coli* isolates.

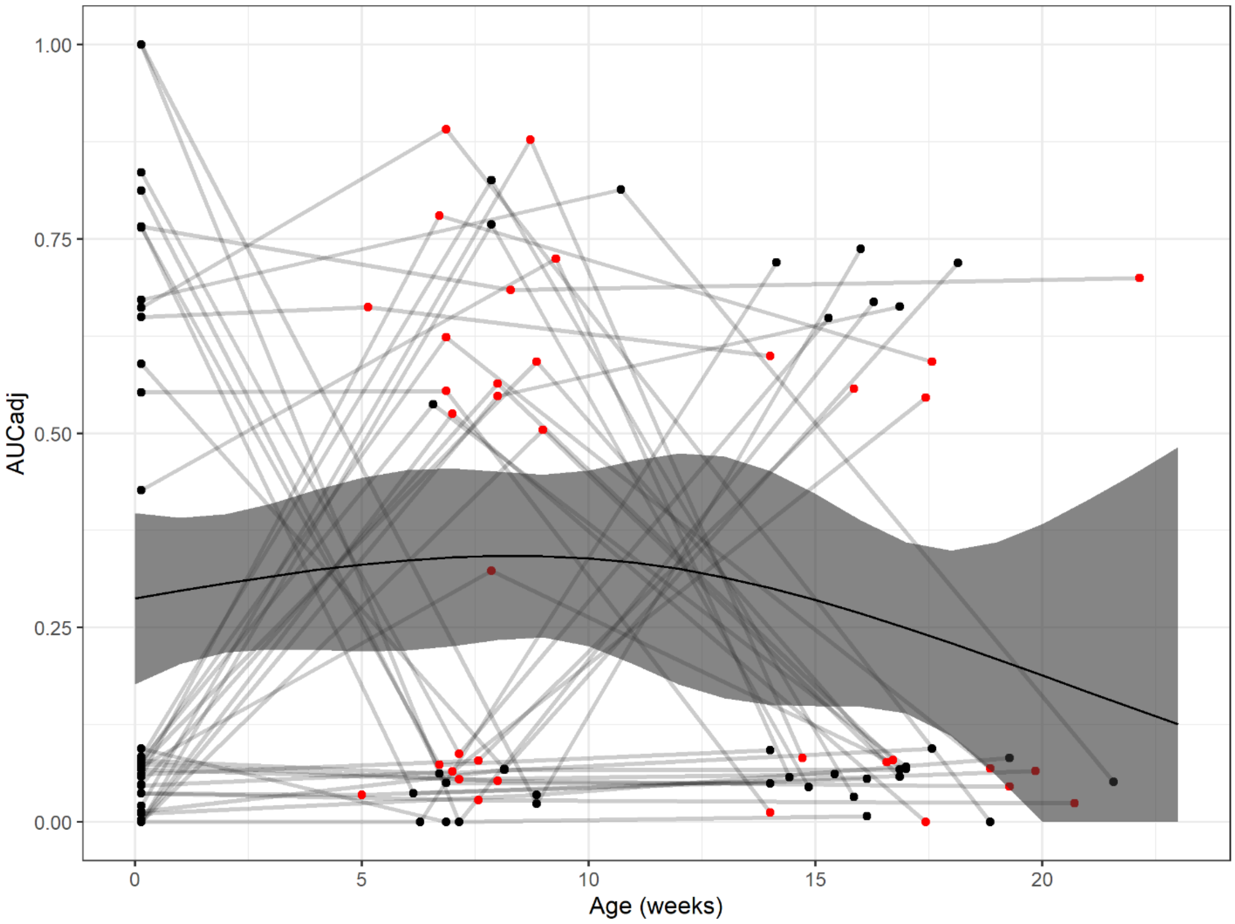

FIG S3. Changes in AUC_{adj} over time (weeks) in chicken flocks, alongside a fitted random effects linear regression model. Red dots indicate use of colistin at any time in the period prior to the sampling; black dots indicate no use of colistin.
